# Bioinformatics Analysis of Stem Cell circ-ASB3 Signaling Pathway and Its Affection on Glioma Biological Characteristics

**DOI:** 10.3389/fninf.2022.859937

**Published:** 2022-04-12

**Authors:** Li Guowei, Jin Yanping

**Affiliations:** ^1^Department of Neurosurgery, The Second Affiliated Hospital of Soochow University, Suzhou, China; ^2^Department of Obstetrics and Gynecology, The Affiliated Suzhou Hospital of Nanjing Medical University, Suzhou, China

**Keywords:** circRNA, bioinformatics analysis, axis, malignancy, glioma stem cell (GSC)

## Abstract

**Objective:**

In our research we try to explore whether glioma stem cell containing circRNAs signal pathway could regulate glioma malignant progression and elaborate its possible mechanism.

**Methods:**

In this study, we used biological information analysis to build an RNA regulatory network and then proceeded RT-PCR to screen target RNAs, after that we clarified the targeting relationship between circRNA-miRNA-mRNA through double luciferase gene assay, RNA pull down experiment, PCR and Western Blot. Finally we adopted RNA transfection to identify its impact on glioma cell proliferation, invasion, migration, apoptosis and cell cycle.

**Results:**

circ-ASB3 was significantly up-regulated in glioma stem cells compared with glioma cells. The circ-ASB3/miR-543/Twist1 axis was discovered to be a possible regulatory pathway in glioma, circ-ASB3 could adsorb and targeted bind to miR-543, down-regulate miR-543 expression, thus release its targeted inhibition to Twist1. Circ-ASB3 was shown to increase glioma cell proliferation, invasion, and migration *in vitro via* miR-543/Twist1 axis. Meanwhile glioma cell apoptosis could be inhibited, and cell cycle arrest could be induced through this signaling pathway.

**Conclusion:**

circ-ASB3 could enhance glioma malignancy *via* miR-543/Twist1 axis, resulting in the discovery of new biomarkers and possible therapeutic targets for these patients.

## Introduction

Eighty-one percent of intracranial malignant tumors are gliomas. Proliferation, dissemination, and invasive tumor growth are the main characteristics of this cancer. Despite advancements in surgical methods, medical imaging technology, adjuvant radiation, chemotherapy, and immunotherapy, the broad diffusion of glioma cells in normal brain parenchyma restricts its therapeutic efficacy (Weller et al., 2010; Ohgaki and Kleihues, 2013; Batich et al., 2017). The median survival time of patients is only 14.6 months, with a 5-year survival rate of <5% (Ostrom et al., 2014; Chang et al., 2015; Barbagallo et al., 2019). The exploration of glioma pathophysiology and new treatment targets have gradually became the focus of current research.

Glioma stem cells (GSCs) are not sensitive to conventional chemoradiotherapy, which mostly kills primary glioma cells. Now some scholars believed that GSC may play important roles in the malignant progression of glioma (Bao et al., 2006). The regulatory mechanism by which GSCs drive tumor progression and recurrence has remained unclear. circRNA, miRNA and mRNA are thought to play a key role in the genesis, development, growth, dissemination, and metastasis of malignant tumors, and previous studied revealed that GSCs contained RNAs showed significant difference compared with glioma cells (Benes and Castoldi, 2010). Moreover, many researchers believed that circRNAs are important in tumor cell cycle, apoptosis, angiogenesis, invasion, and metastasis regulation. In our pre-experiment, we identified the differentially expressed circRNAs in GSCs and found that circ-ASB3 (Hsa_circ_0001005) was significantly up-regulated and may play a critical role in glioma progression.

In this study, we hypothesized that GSCs containing circ-ASB3 could promote glioma progression by regulating the miRNA-mRNA network. Our research tried to confirm the role of circ-ASB3 signal pathway in glioma progression and investigate its underlying mechanism. Our findings revealed that GSCs contained circ-ASB3 could accelerated proliferation, invasion, migration and inhibit apoptosis of glioma cells through miR-543/Twist1 pathway.

## Materials and Methods

### Cell Culture

GSC11 cells were grown in serum-free media (Gibco, USA). Shanghai Institutes for Biological Sciences provided the U87, A172, and U251 cells, which were grown in RPMI 1640 (Gibco, USA) supplemented with 10% fetal bovine serum (FBS), 100 ng/ml streptomycin, and 100 U/ml penicillin (Gibco, USA). All cells were grown at 37°C with 5% CO_2_.

### Differential Gene Identification

The Limma program was used to compare the expression profiles of GSC11 and U87 cells and identify genes that showed significant changes. A value of *P* < 0.05 was indicated statistically significant differences for the data with |LogFC| > 2.

### circRNA-miRNA-mRNA Network Construction

Competitive endogenous RNA (ceRNA), which represents a new regulatory factor, has been the focus of study in recent years. The circInteractome database (https://circinteractome.irp.nia.nih.gov/circular_rna.html) was used to predict circRNA-miRNA interaction pairs. Furthermore, the mid-B, mortar base, and TargetScan databases (http://www.targetscan.org/vert_80/) predicted the interactions between miRNA and mRNA, and the jointly discovered targeted mRNAs were chosen for further investigation. The RNA network was then created by combining circRNA-miRNA interactions with miRNA-mRNA interactions and was visualized using Cytoscape.

### KEGG and GO Analysis

The R program ClusterProfiler was used to identify the differential genes to fully investigate the functional association of these differential RNAs. KEGG (https://www.kegg.jp/kegg/kegg1d.html) and GO (http://geneontology.org) analyses were used to evaluate the relevant accessible categories. There were significant variations when the *P* and Q values were < 0.05.

### RT–PCR

TRIzol reagent was used to isolate total RNA from cells (Invitrogen, USA). The QuantiTect Reverse Transcription Kit was used to generate first-strand cDNA (Qiagen, USA). An ABI 7500 real-time PCR instrument was used to perform PCRs using qPCR SYBR Green Mix (Bio–Rad, USA) (Applied Biosystems, USA). The 2Ct method was used to examine the PCR data, which was performed in triplicate.

### Double Luciferase Assay Report

Cells growing in good condition were cultured in 24-well plates 1 day before plasmid transfection, which was carried out according to the experimental design group. The expression of fluorescence-labeled genes (such as GFP) in cells was observed under fluorescence microscope 24 h after transfection. Afterwards the cells were treated with “Double Luciferase Reporter Gene Detection Kit” (RG027, Biyuntian) and the Luciferase Expression was detected.

### RNA Pull-Down Assay

A172 and U251 cells (1.5 × 10^7^) were collected and lysed using lysis buffer for one night at 4°C and hybridized with specific miR-543 probes for 48 h at 55°C. Biotin-labeled miRNA-543 probes and control probes were purchased from synthesized by RIBOBIO (Guangzhou, China). The cell lysate was incubated with streptavidin agarose beads (Thermo Scientific). TRIzol (Takara, Dalian, China) was used to elute and purify the interacted RNA complex, and qRT-PCR was used to measure circ-ASB3 enrichment pulled down by miR-543 probe.

### Transfection and Cell Therapy

GenePharma provided siRNAs targeting circ-ASB3 and miR-543 inhibitor (Jiangsu, China). These oligonucleotides/vectors were transfected with Lipofectamine 3000® (InvitrogenTM, USA) according to the manufacturer's instructions. Glioma cells were transfected with circ-ASB3 siRNA at a concentration of 20 nM. The knockdown of miR-543 was proceeded through a 20 nM miR-543 inhibitor. While the overexpression of Twist1 was achieved through lentivirus plasmid transfection. The following interventions were performed in each group, NC Group: Si-NC+inhibitor-NC+Vector; Si-circ Group: Si-circ+inh-NC+Vector; Si-circ+inh-miR Group: Si-circ+inh-miR-543+Vector; Si-circ+mRNA Group: Si-circ+inhibitor-NC+Twist1. The transduction method took 24 h to complete. And the transfection efficiency is over 90%.

### Western Blot Analysis

RIPA® buffer (CWBioTM, Beijing, China) was used to lyse the cells. Then, the lysates were placed in loading buffer and denatured at 100°C for 10 min. The resultant solutions were separated by SDS–PAGE before being transferred to PVDF membranes (MilliporeTM, Billerica, Massachusetts, USA). The membranes were placed in an incubator overnight at 4°C with selected primary antibodies (AbcamTM, Cambridge, MA, USA) after a 60-min blocking step. Then, the appropriate secondary antibody was added (CSTTM, Danvers, MA, USA), and the membrane was maintained at room temperature for 120 min. Immobilon Western Chemiluminescent HRP Substrate® (MilliporeTM, USA) was used to assess protein loading. GAPDH was used as a loading control.

### Cell Proliferation Assay

Transfected cells were seeded into 6-well plates and cultured in medium with 10% FBS at 37°C with 50% CO2 for clone creation experiments. Cells were stained with 0.1% crystal violet (BeyotimeTM, Beijing, China) after 10 days of incubation, and colony counts were performed manually.

### Cell Invasion Assay

Cell invasion tests were performed using Matrigel-coated 24-well Transwells (BD, USA). The upper chamber contained 1 × 10^5^ cells in 500 μl of DMEM (1% FBS), and the lower chamber contained 750 μl of DMEM (10% FBS). After 48 h of incubation, the Matrigel and cells in the upper chamber were removed. Then, 4% paraformaldehyde was used to fix cells on the lower surface of the membrane, and 0.5% crystal violet was used to stain the cells. The invasive cells were photographed and quantified in five random fields per well using an inverted microscope (Nikon, Japan).

### Cell Migration Assay

Glioma cells were grown in 6-well plates. After 72 h, serum-free DMEM was added to the plates and incubated for 12 h. All cells were seeded in the designated plates and washed three times with sterile PBS before being scratched (central plate region) with 200 μl pipettor tips. The cells were examined microscopically after 24 h under normal conditions.

### Cell Apoptosis Assay

Glioma cells were collected, washed in PBS, and incubated for 48 h after being analyzed with an Annexin V-FITC Apoptosis Detection Kit® (Beyotime BiotechTM, Haimen, China). Annexin V-FITC was used to label the cells, which were then resuspended in 190 μl of binding buffer before 10 μl of propidium iodide (PI) was added (20 g/ml). Then, the cells were cultured for 15 min in the dark at room temperature before being analyzed by flow cytometry and FACSDiva® software (Version 6.2). The percentages of apoptotic cells within distinct categories were determined after the cell types were separated/grouped into viable, necrotic, and apoptotic cells.

### Cell Cycle Assay

Cells in each group were fixed in 70% ethanol (cold) for 12 h at 4°C. After being washed twice with PBS, the cells were stained with PI in PBS containing RNase. Flow cytometry was then used to assess these treated cells (Beckman Coulter, Inc., Brea, CA, USA).

### Statistical Analysis

Major RNA dysregulations were assessed through R Limma package, data set |LogFC| > 2 and *P* < 0.05 was considered to have significant difference. The R package clusterprofiler was used to annotate the function of differential RNAs in order to comprehensively explore their functional correlation. The GO and KEGG enrichment pathways with *P* and Q values <0.05 were considered as significant categories. The experimental quantified data were shown in Mean±SD with three replications, *T*-test was used to compare the mean difference between two groups. GraphPad Prism 8.0 and SPSS 21.0® software were used for data analysis, *P* < 0.05 was considered as significant difference (^*^P < 0.05, ^**^P < 0.01).

## Results

We aimed to examine the role of GSCs containing circ-ASB3 in glioma progression. First, we conducted bioinformatics analysis and constructed a circRNA-miRNA-mRNA regulatory network. Then, we conducted a series of *in vitro* assays and found that circ-ASB3 could promote glioma progression by regulating miR-543/Twist1 axis. Therefore, our study investigated the functional roles of GSCs containing circ-ASB3 in glioma to provide new insights into the pathogenesis of glioma malignant progression.

### Differential circRNA Identification and Targeted miRNA Prediction

**Figure 1:**
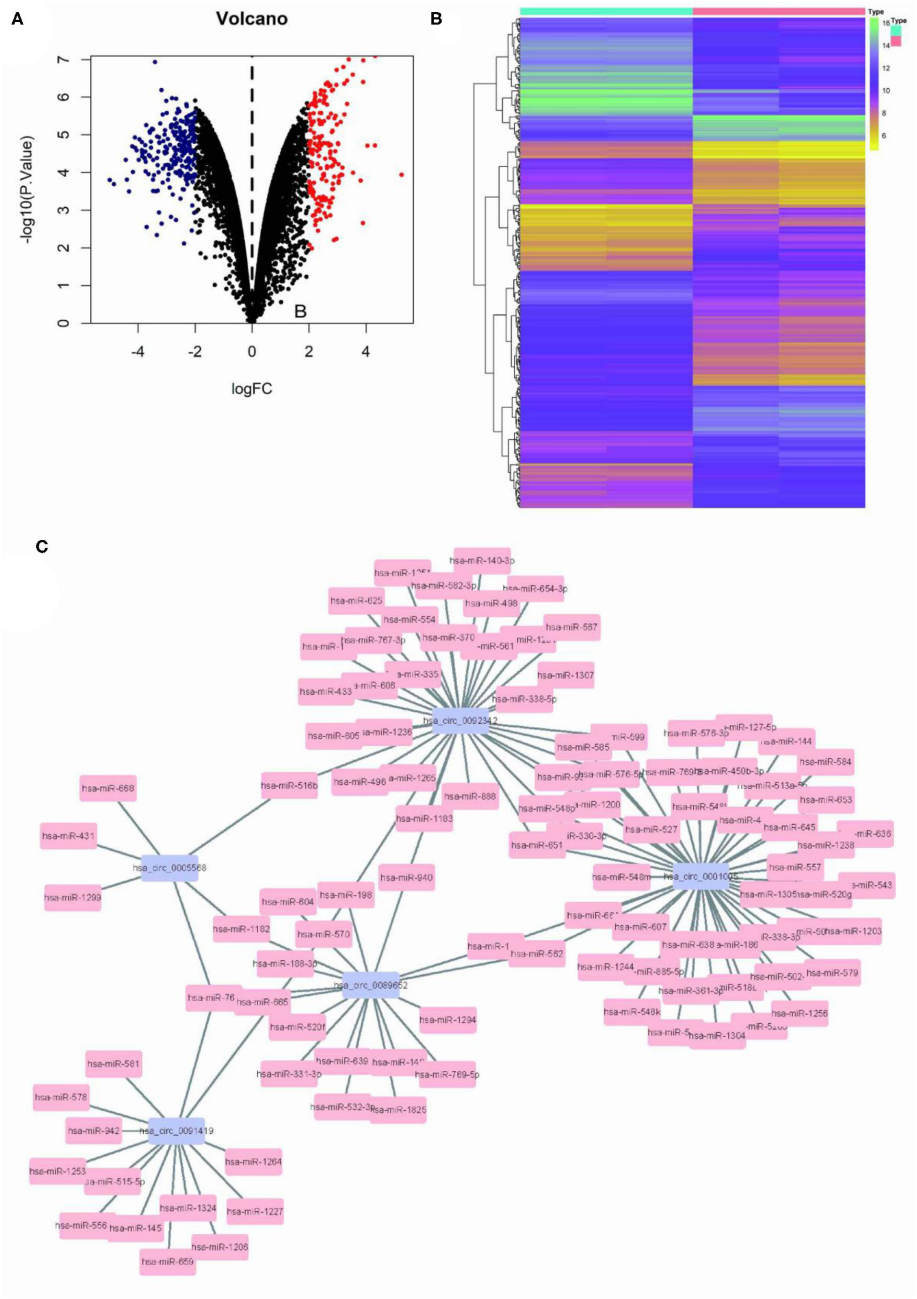
Differential circRNA screening of GSC11 and U87 cells. **(A)** The volcano plot of abnormal circRNA expression in GSC11 cells. **(B)** The heat map of abnormal circRNA expression in GSC11 cells. **(C)** GSC11-highly expressed circRNA-miRNA regulatory networks (125 circRNA-miRNA pairs).

Limma program was used for the differential analysis of circRNA samples (GSC11 vs. U87 cells), and the differential gene screening criteria was set as LOG2FC > 2 and *P* < 0.05, thus screened out 397 differentially expressed circRNAs (174 upregulated and 223 downregulated) (Figures 1A,B). Then, we used the circinteractome database to predict microRNA targets for the top 5 up-regulated circRNAs with the most significant difference. Finally we filtered out a total of 125 circRNA-miRNA interaction pairs (Figure 1C).

### Construction of circRNA-miRNA-mRNA Network

**Figure 2:**
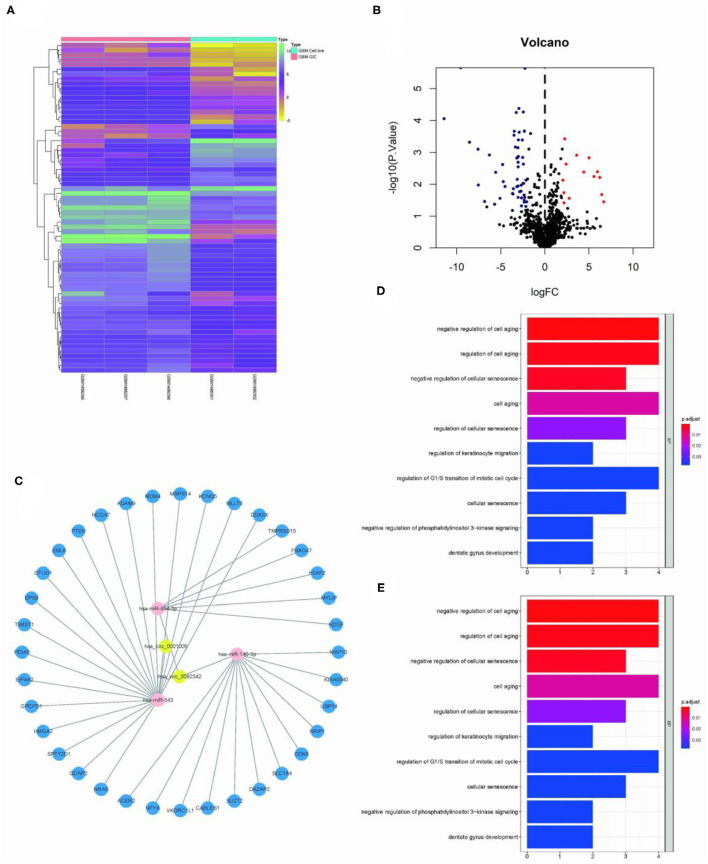
Construction of GSC11-specific miRNA-mRNA regulatory network. **(A,B)** The volcano plot and heat map indicate the aberrant expression of miRNA in GSC11 cells. **(C)** miRNA-mRNA network closely related with highly expressed circRNAs in GSC11 cells has been drawn using Cytoscape (intersect 125 circRNA-miRNA pairs with 55 down-regulated miRNA in GSC11 cells and predict target genes of miRNA through database). **(D)** The primary biological processes and molecular activities closely related with these mRNAs were discovered through GO analysis. **(E)** KEGG pathway enrichment analysis revealed highly enriched cancer-related pathways of these mRNAs.

The Limma package was used for the differential analysis of miRNA samples (GSC vs. GBM), and the differential gene screening condition was LOG2FC > 2 and *P* < 0.05. A total of 69 differentially expressed miRNAs were screened (14 upregulated and 55 downregulated) (Figures 2A,B). We take intersection of these screened 125 circRNA-miRNA pairs with 55 down-regulated miRNAs, thus filtered out 6 circRNA-miRNA pairs. The miRDB?miRTarBase, and TargetScan databases were used to predict miRNA target genes; then 36 RNA relationship pairs were identified. Finally the ceRNA network was successfully established (Figure 2C).

### Functional GO/KEGG Annotation

The ClusterProfiler package was used for functional enrichment analysis of these 36 mRNAs; GO analysis revealed that gene enrichment pathways mostly focused on the positive control of cell aging and the negative regulation of cellular senescence. According to KEGG analysis, these mRNAs were mostly found to be closely related with cancer miRNAs, p53 signaling pathways, and glioma (Figures 2D,E).

### circ-ASB3 Sponged miR-543 and Up-Regulated Twist1 Expression

**Figure 3:**
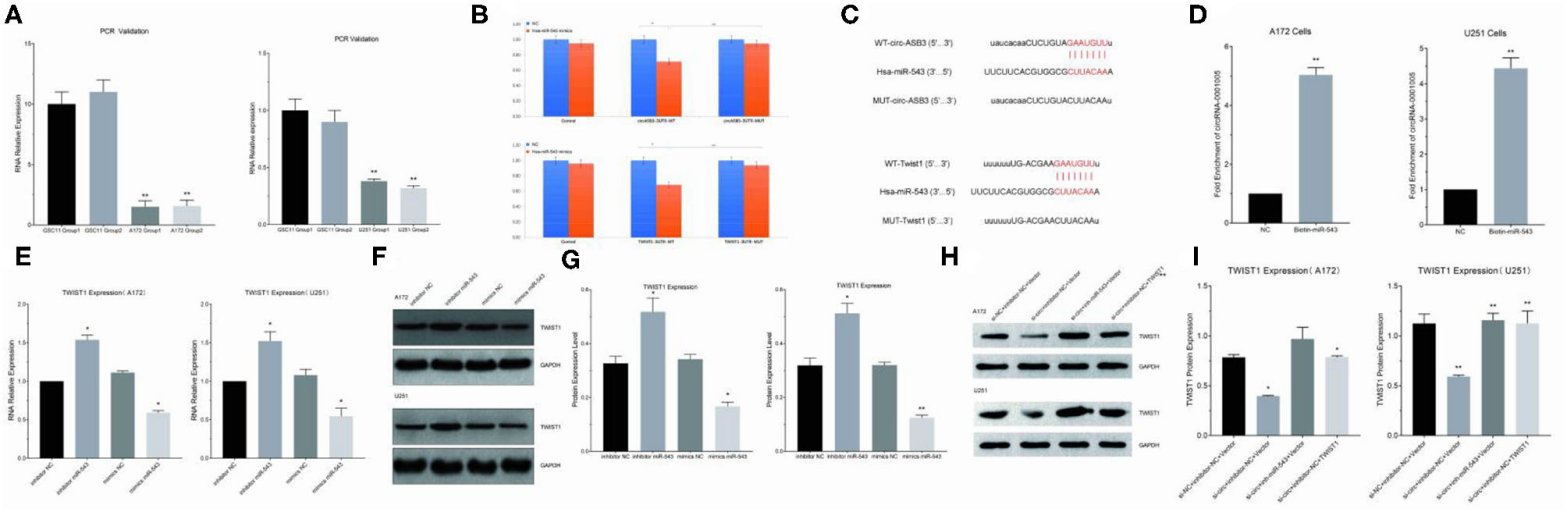
Circ-ASB3 up-regulated Twist1 expression by sponging mR-543. **(A)** RT-PCR was used to detect relative expression of circ-ASB3 in GSC11 cells (Compared with A172 and U251). **(B)** Luciferase reporter activity of circ-ASB3/Twist1 in A172 cells was analyzed after co-transfected with miR-543 mimic and mimic control. **(C)** Schematic representation of circ-ASB3/Twist1 3′-UTR in wild type and circ-ASB3/Twist1 3′-UTR in mutant type, with mutations at the predicted miR-543 binding site. **(D)** RNA pull-down assay after transfection of biotin-labeled oligo and miR-543 probes in A172 cells. **(E)** RT-PCR detection of Twist1 mRNA in A172 cells after transfected with miR-543 inhibitor and mimics. **(F,G)** Twist1 expression was identified using western blot assays after transfected with miR-543 inhibitor and mimics. **(H,I)** Twist1 expression was identified using western blot assays after transfected with si-circ-ASB3, miR-543 inhibitor and Twist1 mimics. The data are the MEAN±SEM of three experiments, ^*^P < 0.05, ^**^P < 0.01; representative images are presented.

Circ-ASB3 was significantly upregulated in GSC11 cells compared with their levels in A172 and U251 cells (Figure 3A). Through bioinformatics analysis we revealed that circ-ASB3 and Twist1 contain a putative binding site of miR-543 (Figure 3C). Thus we constructed plasmids containing WT or mutated type (MUT) miR-543 binding site and then subcloned them into the pGL3 Basic reporter vectors. After that we co-transfected these vectors into A172 cells with pGL3-circ-ASB3/Twist1-MUT, pGL3-circ-ASB3/Twist1-WT and matched controls. Cell collection was proceeded 24 h after transfection and Dual-Luciferase Assay System was adopted for analysis.

The results showed that the activity of circ-ASB3-3UTR region decreased by 29% with the effect of miR-543 (*P* < 0.01), indicating that miR-543 could have impact on circ-ASB3-3UTR region, the activity of circ-ASB3-3UTR region increased by 32% with the mutating of circ-ASB3-3UTR region (*P* < 0.05), indicating that this mutation site is the interaction region of circ-ASB3/miR-543; meanwhile the activity of Twist1-3UTR region decreased by 32% compared with control group (*P* < 0.01), indicating that miR-543 could affect Twist1-3UTR region, the activity of Twist1-3UTR region increased by 37% with the mutating of Twist1-3UTR region (*P* < 0.01), indicating that this mutation site is the interaction region of miR-543/Twist1 (Figure 3B).

In addition, circRNA pull-down assays were performed to validate the direct binding of circ-ASB3 and miR-543 in glioma cells. In this experiment significant enrichment of circ-ASB3 was confirmed by qRT-PCR in miR-543 probe group, and this enrichment of circ-ASB3 in biotin-labeled miR-543 further proved the interaction between circ-ASB3 and miR-543 (Figure 3D).

Through RNA transfection, RT-PCR and Western Blot Assays, we found that circ-ASB3 down-regulation could inhibit Twist1 expression, while miR-543 inhibition could reverse this effect (Figures 3H,I), at the same time miR-543 inhibition could down-regulate Twist1 expression, miR-543 overexpression could achieve the opposite effect (Figures 3E–G). Overall, these findings showed that circ-ASB3 acts as a sponge to up-regulate Twist1 expression by targeting miR-543.

### circ-ASB3 Accelerated Glioma Proliferation, Invasion, and Migration Through miR-543/Twist1 Axis

**Figure 4:**
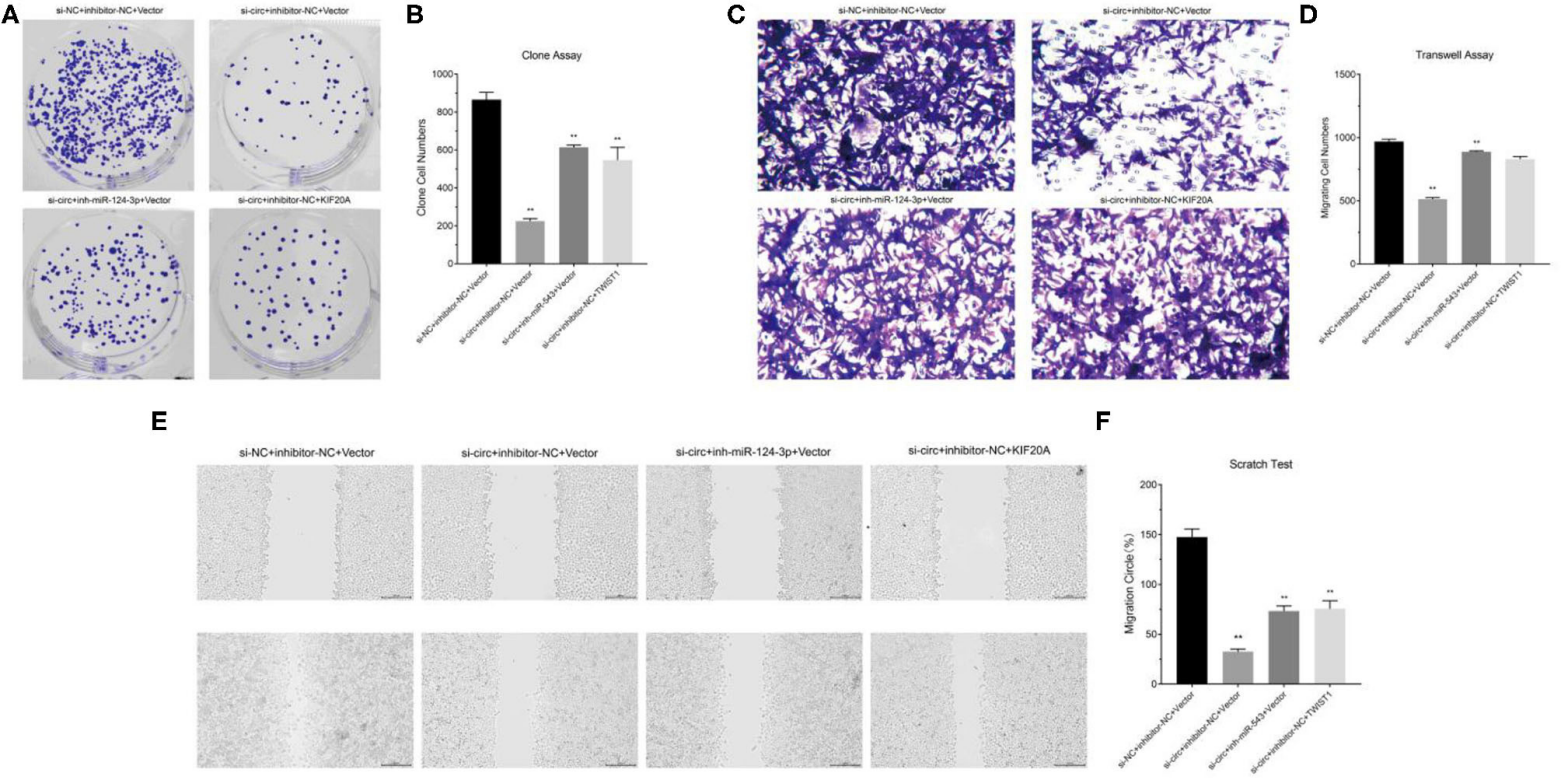
Circ-ASB3 accelerated glioma proliferation, invasion and migration through miR-543/Twist1 axis. **(A,B)** Colony formation assays detected the cell proliferation ability of A172 cells transfected with si-circ-ASB3, miR-543 inhibitor and Twist1 mimics. **(C,D)** Transwell assays measured the invasion abilities of A172 cells transfected with si-circ-ASB3, miR-543 inhibitor and Twist1 mimics. **(E,F)** The migration ability of A172 cells transfected with si-circ-ASB3, miR-543 inhibitor and Twist1 mimics was measured through scratch test (0 and 24 h), ^**^P < 0.01; representative images are presented.

We constructed cell models of different RNA expression through plasmid transfection and detected the functional changes in glioma cells. The results of clone formation assay showed that in circ-ASB3 down-regulated glioma cells, the number of new cells obviously decreased, while in glioma cells combined with combined with miR-543 inhibition or Twist1 overexpression, the number of new cells correspondingly increased (Figures 4A,B). The results of invasion assay showed that in circ-ASB3 down-regulated glioma cells the vertically-migrated cells decreased significantly, while in glioma cells combined with miR-543 inhibition or Twist1 overexpression, the number of vertically-migrated cells correspondingly increased (Figures 4C,D). The results of migration assay showed that in circ-ASB3 down-regulated glioma cells the transversely-migrated cells evidently decreased, while in glioma cells combined with miR-543 inhibition or Twist1 overexpression, the number of transversely-migrated cells correspondingly increased (Figures 4E,F). These results suggested that down-regulated circ-ASB3 could inhibit glioma cell proliferation, invasion and migration while miR-543 down-regulation and Twist1 up-regulation could reverse these changes.

### circ-ASB3 Inhibited Glioma Apoptosis and Induced Cell Cycle Arrest Through miR-543/Twist1 Axis

**Figure 5:**
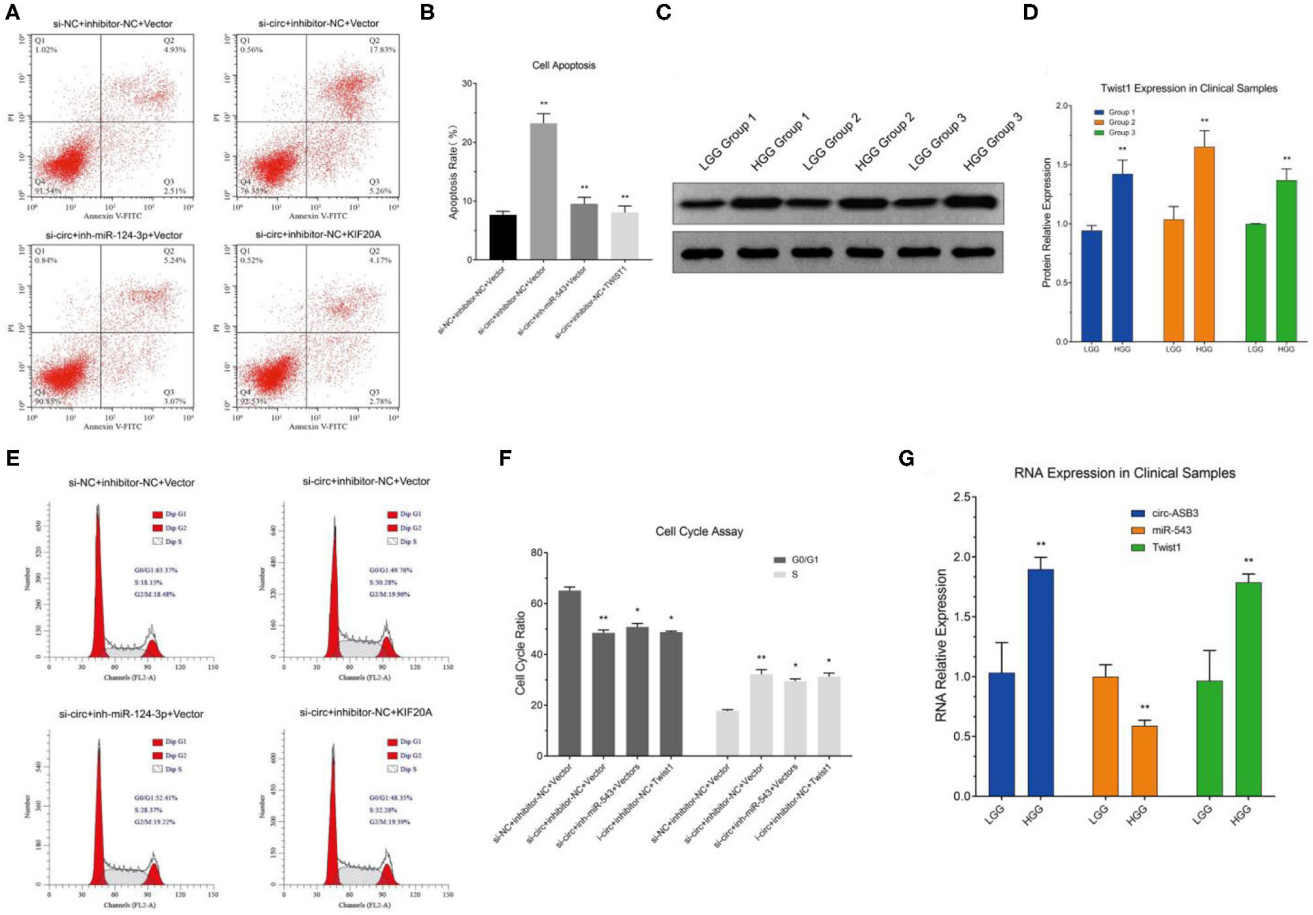
Circ-ASB3 inhibited glioma apoptosis and induced cell cycle arrest through miR-543/Twist1 axis, clinical sample validation. **(A,B)** Apoptosis rate was detected by Annexin V-FITC staining of A172 cells transfected with si-circ-ASB3, miR-543 inhibitor and Twist1 mimics. **(E,F)** The cell cycle of A172 cells transfected with si-circ-ASB3, miR-543 inhibitor and Twist1 mimics was examined by Flow cytometry. **(G)** PCR detection of target signal pathway RNA in clinical samples. **(C,D)** Western Blot analysis of Twist1 protein content in clinical samples, ^*^P < 0.05, ^**^P < 0.01; representative images are presented.

According to the results of apoptosis detection, in circ-ASB3 down-regulated glioma cells the percentage of apoptotic cells increased significantly, while in glioma cells combined with miR-543 inhibition or Twist1 overexpression, the percentage of apoptotic cells correspondingly decreased (Figures 5A,B). The cell cycle detection revealed that in circ-ASB3 down-regulated glioma cells, the percentage of cells in G0/G1 phase decreased and percentage of cells in S phase increased, while in glioma cells combined with miR-543 inhibition or Twist1 overexpression, the percentage of cells in G0/G1 phase correspondingly increased and percentage of cells in S phase correspondingly decreased (Figures 5E,F). These results indicated that down-regulated circ-ASB3 could promote glioma cell apoptosis and cell cycle, while miR-543 down-regulation and Twist1 up-regulation could reverse these changes.

### RNA and Protein Expression Detection of Signal Pathway in Clinical Samples

We selected 53 clinical samples and divided them into Low Grade group (15 cases) and High Grade group (38 cases) (Low Grade: I-II, High Grade: III-IV) according to pathology laboratory. The signal pathway RNA and protein expression detection revealed that the circ-ASB3 and Twist1 gene expressions were significantly up-regulated while miR-543 gene expression was significantly down-regulated in high-grade glioma samples (Figure 5G). In the meantime, Twist1 protein content was obviously increased in high grade glioma samples (Figures 5C,D). These verifications were consistent with the results of *in vitro* experiments.

## Discussion

Most abnormally expressed miRNAs appeared to act as oncogenes and were implicated in the malignant transformation of glioma. The EGFR, PI3K/Akt, p53, TGF-dependent apoptotic signaling cascades, Notch and NF-κB pathways were among the most important pathways, while miRNAs played a key role in regulating these pathways, indicating a close relationship with distinct malignant glioma morphologies (Novakova et al., 2009; Zhang et al., 2009; Zhou et al., 2010; Li et al., 2015; Cao et al., 2019; Deng et al., 2020).

Previous studies have shown that circRNAs may play important regulatory roles in the tumorigenesis, malignant progression and recurrence of glioma. For example, circ-0005198 plays a role in glioma proliferation, invasion, migration and apoptosis. Fzd7 acted as a miR-638 regulating factors, could be regulated by hsa-circ-0000177, and finally stimulated the Wnt signaling pathway to regulate glioma growth (Burd et al., 2010; Du et al., 2017; Li et al., 2018).

Previous research suggested that GSCs may be closely related to malignant progression and chemoradiotherapy resistance of glioma cells. For instance ATP binding cassette drug transporters (ABC) in GSCs could transport intracellular chemotherapeutic drugs to the outside of cells, thus to reduce its damage to GSCs in chemotherapy procedures; on the other hand, the repair efficiency of damaged DNA in GSCs cells is apparently higher than glioma cells. This mechanism may play important roles in the chemoradiotherapy resistance of glioma (Clarke et al., 2006; Huang et al., 2010; Chan et al., 2012). However, until now the key regulatory factors and the regulatory mechanism of GSCs on malignant glioma cells have not been fully clarified. It is now generally accepted that breaking the regulatory network between GSCs and glioma cells is crucial to disturb the malignant pathological progression of glioma. Our research tried to construct the RNA regulatory network between GSCs and glioma cells, meanwhile we also illustrated its regulation mechanism through *in vitro* experiments.

In this study, bioinformatics analysis was used to screen the expression profiles of GSCs and glioma cells for tumorigenic RNAs. First we proceeded circRNA sequencing between GSC11 and U87 cells, then we used microarray data and Limma program for screening differentially expressed circRNAs. The circRNA-miRNA prediction was performed with the circinteractome database, while the miRNA target gene prediction was performed through miRDB, miRTarBase and TargetScan databases. Finally, the circRNA-miRNA-mRNA regulatory network was constructed (including 2 circRNAs, 3 miRNAs, and 36 mRNAs). GO/KEGG functional enrichment analyses revealed that these mRNAs may be closely related to cancer associated miRNAs, p53 signaling pathway and glioma. Afterwards the candidate circRNAs were verified by PCR validation. The results indicated that circ-ASB3 may be involved in the regulation of glioma pathological process.

Zhang discovered that the circ-ASB3 inhibition could increase mitochondrial apoptosis and autophagy, which could accelerate cell death in hepatocellular carcinoma cells. Other researchers revealed that circ-ASB3 could modulate caspase-8-mediated Beclin1 cleavage and thus regulate mitochondrial apoptosis pathway (Zhang et al., 2019).

Previous studies revealed that miR-543 expression was obviously down-regulated in glioma, and functional investigations showed that it could induce cell apoptosis and reduce cell proliferation, invasion and migration. Furthermore, *in vivo* study revealed that miR-543 could inhibit the tumorigenicity of glioma cells (Haga and Phinney, 2012; Fan et al., 2016).

Twist1 is a master regulator of epithelial mesenchymal transition (EMT) and a bHLH transcription factor (TF). Cheng and Zhang reported that Twist1 over-expression could enhance tumor cell proliferation and migration. Mikheeva showed that Twist1 could promote glioblastoma multiforme invasion by inducing mesenchymal transition without classic cadherin flipping, meanwhile Twist1 over-expression may block p53 expression. On the contrary the absence of Twist1 usually result in a high rate of apoptosis, cell cycle arrest in G0/G1 phase and proliferation inhibition (Mikheeva et al., 2010; Qiu et al., 2020).

By constructing GSCs specific circRNA-miRNA-mRNA regulatory network, exploring mRNA function, consulting relevant literatures and proceeding RT-PCR verification, we found that circ-ASB3/miR-543/Twist1 signal pathway may be closely related to the regulation of glioma biological characteristics. Meanwhile increasing evidence have suggested that circRNA could act as a sponge of miRNAs to regulate RNA expressions and protein functions, thus we further assumed that circ-ASB3 may competitively inhibit miR-543, and finally effected the Twist1 expression, thus promoting the malignant progression of glioma.

By means of double luciferase gene assay and RNA pull down we found that there is a direct targeting relationship between circ-ASB3 vs. miR-543/miR-543 vs. Twist1, in the meantime we also illuminated that circ-ASB3 could targeted binding to miR-543 in chr2:53992534-53992556 (CUUACAA), while Twist could targeted binding to miR-543 in chr7:19155470-19155490 (CUUACAA). Through RNA transfection, PCR and Western Blot detections, we identified that miR-543 could inhibit Twist1 expression, while circ-ASB3 could competitively inhibit miR-543, thus relieve its suppression on Twist1 expression.

Moreover, through cell culture and RNA transfection we discovered that the down-regulation of circ-ASB3 could inhibit glioma proliferation, invasion and migration, meanwhile this down-regulation could also promote cell apoptosis and accelerate cell cycle. Through downstream RNA recovery we confirmed that these effects could be rescued by miRNA-543 inhibitor or Twist1 mimics. All these findings suggested that circ-ASB3 may enhance glioma malignant progression through miR-543/Twist1 axis.

In previous relevant studies, most scholars focused on parenchymal tumor cells, our experiment put aside the conventional ideas of glioma research, and creatively selected GSCs as the entry point, proposed that GSCs may play a key role in glioma malignant progression, thus introduced stem cell theory into the elaboration of glioma related regulatory mechanism.

In target pathway screening section we abandoned the common mentality of combining theoretical assumption with literature review to predict signal pathway, and introduced bioinformatics analysis to construct RNA regulation networks, used big data to accurately screen and locate target genes and regulation pathways. Thus avoid the unknowness, contingency and limitations of our research objects, therefore our results and conclusions are more convincing.

## Conclusion

In summary, circ-ASB3 was significantly up-regulated in glioma stem cells, it could increase Twist1 expression by competitively inhibiting miR-543, thus promoting glioma malignant transformation. Our study clarified the role of GSCs in the malignant progression of glioma, providing new ideas and methods to explore the regulatory mechanism of glioma malignant progression. Finally our results revealed that circ-ASB3 could act as a new biomarker associated with the malignant progression of glioma and offer a potential therapeutic target for these patients.

## Data Availability Statement

The original contributions presented in the study are included in the article/supplementary material, further inquiries can be directed to the corresponding author.

## Author Contributions

JY and LG provided the design idea of this study. LG drafted the manuscript. JY revised the manuscript. Both authors read and approved the final manuscript.

## Conflict of Interest

The authors declare that the research was conducted in the absence of any commercial or financial relationships that could be construed as a potential conflict of interest.

## Publisher's Note

All claims expressed in this article are solely those of the authors and do not necessarily represent those of their affiliated organizations, or those of the publisher, the editors and the reviewers. Any product that may be evaluated in this article, or claim that may be made by its manufacturer, is not guaranteed or endorsed by the publisher.
